# Prolonged Neuromodulation of Cortical Networks Following Low-Frequency rTMS and Its Potential for Clinical Interventions

**DOI:** 10.3389/fpsyg.2019.00529

**Published:** 2019-03-12

**Authors:** Grace Edwards, Sara Agosta, Florian Herpich, Federica Contò, Danielle Parrott, Sarah Tyler, Emily D. Grossman, Lorella Battelli

**Affiliations:** ^1^Center for Neuroscience and Cognitive Systems@UniTn, Istituto Italiano di Tecnologia, Rovereto, Italy; ^2^Department of Psychology, Harvard University, Cambridge, MA, United States; ^3^Center for Mind/Brain Sciences, University of Trento, Trento, Italy; ^4^Department of Psychology, University of California, San Diego, La Jolla, CA, United States; ^5^Department of Cognitive Sciences, University of California, Irvine, Irvine, CA, United States; ^6^Department of Neurology, Berenson-Allen Center for Noninvasive Brain Stimulation, Beth Israel Deaconess Medical Center, Harvard Medical School, Boston, MA, United States

**Keywords:** non-invasive brain stimulation, low frequency (1 Hz) repetitive transcranial magnetic stimulation, clinical intervention, transcranial electric stimulation, prolonged neuromodulation

## Abstract

Non-invasive brain stimulation safely induces persistent large-scale neural modulation in functionally connected brain circuits. Interruption models of repetitive transcranial magnetic stimulation (rTMS) capitalize on the acute impact of brain stimulation, which decays over minutes. However, rTMS also induces longer-lasting impact on cortical functions, evident by the use of multi-session rTMS in clinical population for therapeutic purposes. Defining the persistent cortical dynamics induced by rTMS is complicated by the complex balance of excitation and inhibition among functionally connected networks. Nonetheless, it is these neuronal dynamic responses that are essential for the development of new neuromodulatory protocols for translational applications. We will review evidence of prolonged changes of cortical response, tens of minutes following one session of low frequency rTMS over the cortex. We will focus on the different methods which resulted in prolonged behavioral and brain changes, such as the combination of brain stimulation techniques, and individually tailored stimulation protocols. We will also highlight studies which apply these methods in multi-session stimulation practices to extend stimulation impact into weeks and months. Our data and others’ indicate that *delayed* cortical dynamics may persist much longer than previously thought and have potential as an extended temporal window during which cortical plasticity may be enhanced.

## Introduction

Repetitive transcranial magnetic stimulation (rTMS) has been increasingly used in the last 20 years to align cortical regions to cognitive function (Chen et al., 1997; Walsh and Cowey, 2000). The impact of neuromodulation on targeted neural mechanisms and the concomitant behavioral function depends critically on the delivery and protocol of stimulation. Whereas high-frequency rTMS (HF-rTMS) is associated with increased cortical excitability (Pascual-Leone et al., 1994), low-frequency rTMS (LF-rTMS) is an inhibitory protocol that results in acute temporary impairments in function mediated by the stimulated brain region (Chen et al., 1997), and interconnected areas (Grefkes et al., 2010; Lee and D’Esposito, 2012; Plow et al., 2014; Battelli et al., 2017). For example, LF-rTMS to the intraparietal sulcus (IPS) causes a decrease in sustained attention (e.g., tracking multiple moving objects) in the visual field contralateral to stimulation, indicating the necessary role of the IPS in spatial attention (Battelli et al., 2009; Edwards et al., 2017).

The immediate effects of neuromodulation typically outlast the duration of stimulation (Thut and Pascual-Leone, 2010), and thus may be a marker of enduring plasticity. Physiologically, TMS-related excitatory/inhibitory effects have been associated with long term potentiation and depression mechanisms, respectively (LTP/LTD, Thickbroom, 2007). While the behavioral effect is short lived, the stimulation effects upon physiology, expressed as a delayed change in functional connectivity among nodes of the attention network, persist much longer, indicating late developing metaplastic changes (Sporns et al., 2004; Thickbroom, 2007; Battelli et al., 2017) In recent years, characterizations of the underlying mechanisms following stimulation and accumulating evidence of behavioral and brain modulation beyond the initial phase after stimulation have opened the field to the potential for rTMS to promote enduring plasticity (Ridding and Rothwell, 2007).

Prolonged neuromodulation following LF-rTMS has received less attention than acute stimulation effects, however, durable LF-rTMS interventions have great potential as a therapeutic aid (Hallett, 2007; Raffin and Siebner, 2014; Silasi and Murphy, 2014). rTMS can be readily paired with neurophysiological and psychophysical measures to evaluate the persistent cortical dynamics, and their potential behavioral correlates following brain stimulation. The expected scientific gains are not trivial: knowledge of brain and behavior fluctuations for sustained periods of time following stimulation allow for more statistically robust experimental designs and, crucially, for better experimental and clinical protocols. Further, recording beyond the initial phase following stimulation will bridge models of acute changes in function with sustained, translational intervention approaches.

Here we review the prolonged effects of LF-rTMS, highlighting protocols used to increase durability across hours to days and months. Prolonged duration of neuromodulation following LF-rTMS has strong clinical potential and is yet to be highlighted as thoroughly as those of high-frequency stimulation protocols (Schutter, 2009; Berlim et al., 2013; Lefaucheur et al., 2014). We will also feature the variables that interact with brain stimulation to boost or alter predicted stimulation outcome. Two lines of stimulation protocols will be considered: (1) the long-term post-stimulation effects from one session of stimulation, and (2) the summation effects of multi-sessions. Durability from a single session is likely to translate to endurance across sessions, yet single- and multi-session protocols have been thus far studied independently. For details on each stimulation protocol which resulted in prolonged stimulation effects, see Table 1.

**Table 1:** Summary of study protocols and prolonged stimulation effects.

Author	Target	Protocol	Stimulation duration	Post-stimulation effects	Measure	Effect direction	Subjects (*n*)
**Studies with prolonged stimulation effects following LF-rTMS**							
Agosta et al., 2014	P3 (left parietal cortex)	LF-rTMS (1 Hz) 90% MT	10 min	30 min	Bilateral multiple object tracking	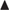 Left visual field	Right hemi lesion patients (6)/controls (6)
Battelli et al., 2017	Left posterior IPS	LF-rTMS (1 Hz) 75% MO	15 min	50 min	Functional connectivity	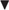 / 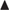	Human (9)
Brighina et al., 2003	P5 (left parietal cortex)	LF-rTMS (1 Hz) 90% MT	15 min/ seven sessions (over 2 weeks)	15 days	Visuospatial performance	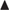 Left visual field	Right hemi lesion patients (3)/controls (5)
Demirtas-Tatlidede et al., 2015	Primary motor cortex	LF-rTMS (1 Hz) 100% MT	20 min daily/10 weekdays	4 weeks	Fractional anisotropy and motor eval.	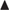	Chronic stroke patients (10)
Fregni et al., 2006	Primary motor cortex	LF-rTMS (1 Hz) 100% MT	20 min daily/5 days	14 days	Clinical motor evaluations	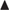 Affected hand	Chronic stroke patients (15)
Stern et al., 2007	rDLPFC	LF-rTMS (1 Hz) 110% MT	20 min/10 days (in 2 weeks)	2 weeks	Hamilton Depression Rating Scale (21-HAM-D)	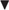	Patients (10)/Sham (5)
Iyer et al., 2003	Motor cortex	HF-rTMS (6 Hz) prime 90% MT → LF-rTMS (1 Hz) 115% MT	10 min → 10 min	60 min	Motor-evoked potential amplitude	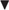	Human (26)
Khedr et al., 2005	Motor cortex (stroke affected)	LF-rTMS (intermittent-3 Hz) 120% MT	6 min daily/10 sessions	10 days	Clinical motor evaluations	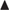 Affected hand	Stroke patients (52)
Muller et al., 2014	Motor cortex	LF-rTMS (1 Hz) 100% MT	5 min	47 min	Motor-evoked potential amplitude	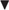	Rats (48)
Naeser et al., 2005	Right broca homolog	LF-rTMS (1 Hz) 90% MT	20 min/10 days (in 2 weeks)	8 months	Standardized language tests	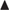	Aphasia patients (4)
Schutter et al., 2001	rDLPFC	LF-rTMS (1 Hz) 130% MT	20 min	65 min	Theta-power/behavioral rating	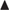 Theta/ 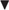 anxiety	Human (12)
Siebner et al., 2004	Primary motor cortex	a/c-tDCS prime 1 mA → LF-rTMS (1 Hz) 90% MT	10 min → 15 min	20 min	Motor-evoked potential amplitude	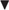 Anodal/ 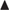 cathodal	Human (8)
Valero-Cabré et al., 2007	Visual parietal cortex	LF-rTMS (1 Hz) 135% MT	30 min	60 min	Metabolic activity (14C-2DG uptake)	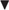	Cats (10)
**Other stimulation protocols resulting in prolonged stimulation effects**							
Gersner et al., 2011	Frontal cortex	HF-rTMS (intermittent-20 Hz) 120% MT	9 min/10 sessions (in 2 weeks)	3 days	Neuroplasticity markers	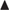 Awake/ 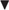 anesthetized	Awake and anesthetized rats (68)
Hoppenrath and Funke, 2013	Layers 2/3 cortex wide	HF-rTMS (intermittent-TBS) 23% MO	192 s	160 min	Cortical proteins (inhib. and excit. markers)	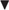 Inhib./ 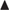 excite.	Rat (42)
Nyffeler et al., 2006	FEF	HF-rTMS (continuous-TBS) 80% MT	33 s	30 min	Saccade latency	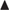	Human (3)
Nyffeler et al., 2009	P3 (left parietal cortex)	HF-rTMS (continuous-TBS) 100% MT	44 s	32 h	Peripheral visual attention	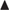 Left visual field	Right hemi. lesion patients (11)
Kasten et al., 2016	Cz/Oz (occipital cortex)	tACS (individual alpha frequency) 1.2 mA	20 min	70 min	EEG	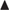 Alpha power	Human (22)
Cappelletti et al., 2013	P3/P4 (parietal cortex)	HF-tRNS 1 mA with behavioral training	20 min daily/5 days	16 weeks	Numerosity discrimination	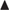	Human (40)
Herpich et al., 2018	O1/O2 (occipital cortex)	HF-tRNS 1 mA	20 min	60 min	Phosphene threshold	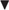	Human (18)
Terney et al., 2008	Motor cortex	HF-tRNS 1 mA	10 min	60 min	Motor-evoked potential amplitude	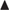	Human (80)
Snowball et al., 2013	F3/F4 (DLPFC)	HF-tRNS 1 mA	20 min daily/5 days	6 months	Near infrared spectroscopy/ Arithmetic	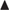 Efficient coupling/ 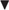 behavior	Human (25)
Romei et al., 2016a	V1—-V5	cc-PAS (0.1 Hz) 70% MO	15 min	60 min	Visual motion sensitivity	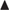	Human (32)
Kuo et al., 2013	Motor cortex	a/c-tDCS (high-def) 2 mA	10 min	120 min	Motor cortex excitability	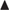 Anodal/ 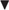 cathodal	Human (14)


*rTMS, repetitive transcranial magnetic stimulation; LF-, low-frequency; HF-, high-frequency; a/c-tDCS, anodal or cathodal transcranial direct current stimulation; tACS, transcranial alternating current stimulation; tRNS, transcranial random noise stimulation; TBS, theta-burst stimulation; cc-PAS, cortico-cortical paired pulse stimulation; MO, machine output; MT, motor threshold; P3, P5, F3, F4, 10–20 electroencephalography electrode placement; IPS, intraparietal sulcus; rDLPFC, right dorsal lateral prefrontal cortex; FEF, frontal eye fields; V1, V5, visual cortex; EEG, electroencephalography; 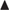, increased effect; 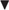, decreased effect*.

## Prolonged LF-rTMS Effects From One Session

Low-frequency rTMS is known to have the potential to modify behavior for a duration that last approximately as long as the stimulation interval itself (Chen et al., 1997). These behavioral interventions are reflected in cortical changes at both the systems and cellular levels (see Raffin and Siebner, 2014; Tang et al., 2017; Hartwigsen, 2018). Animal models of the neurosynaptic mechanisms confirm that rTMS induces altered synaptic efficacy comparable to plasticity mediated through LTD or LTP (Thickbroom, 2007; Vlachos et al., 2012; Muller et al., 2014; Lenz et al., 2016). The frequency dependencies of the experience-dependent plasticity mechanisms are believed to be the basis of frequency dependent facilitation and inhibition from HF-rTMS and LF-rTMS, respectively (Hallett, 2007).

In some LF-rTMS protocols, the impact of stimulation on behavior may extend well beyond the duration of the stimulation (Thut and Pascual-Leone, 2010). For example, 5 min of inhibitory LF-rTMS to mice motor cortex reduces the motor response (as measured with motor evoked potentials, MEP) for more than 45 min following stimulation. Interestingly, the inhibitory effect is prevented if TMS is delivered with receptor-dependent LTD antagonists (Muller et al., 2014). rTMS has potential to elicit a cascade of biophysical changes which extend well beyond the acute period after stimulation which is consistent with evidence for distinct cellular mechanisms underlying LTP-LTD at a range of timescales (Raymond, 2007; Pell et al., 2011).

The offline “perturb-and-measure” approach is also conducive to combined LF-rTMS with neurophysiology. It is from these combined methodological studies that documented lasting effects from one session of LF-rTMS on sustained neural activity (Siebner et al., 2009; Thut and Pascual-Leone, 2010). For example, Schutter et al. (2001) identified increased theta power (a suggested neuromarker for reduced anxiety, Panksepp, 2004) sustained across three recordings up to 65 min after 20 min of LF-rTMS. The increased theta was coupled with behavioral reports of a reduction in anxiety. Using the offline rTMS, studies focused on the distal, network-wide stimulation effects have also produced lasting behavioral change (Nyffeler et al., 2009; Agosta et al., 2014). This is crucial if one ought to use LF-rTMS protocols in the clinical population to help recovery from stroke. Using LF-rTMS to the healthy parietal cortex of unilateral stroke patients, Agosta et al. (2014) suppressed unilateral visual neglect symptoms in the neglected visual field for 30 min following stimulation. This is likely a result of relief from the excess inhibition from the healthy hemisphere upon the lesioned one in chronic stroke (Kinsbourne, 1977; Silasi and Murphy, 2014). Previous work using the more intensive rTMS protocol of continuous theta burst on the healthy hemisphere also resulted in lasting attentional improvement in the neglect field (Nyffeler et al., 2009). These results demonstrate that network-wide stimulation effects can outlast the acute effects regularly reported.

**FIGURE 1:**
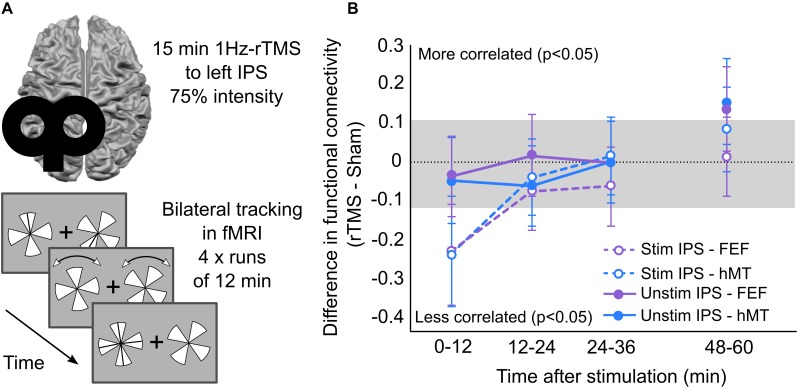
Lasting functional connectivity changes in dorsal attention network following LF-rTMS to left IPS. **(A)** Battelli et al. (2017) methods: participants received 1 Hz rTMS applied to left IPS for 15 min at 75% intensity. Following stimulation participants perform bilateral tracking paradigm in the fMRI for 1 h. In the task, two pinwheels are presented either side of fixation cross. One section of each pinwheel is marked to be tracked using a line. The line disappears, and the pinwheels rotate bi-directionally with random changes in direction. When the pinwheels stop moving, one pinwheel is highlighted for the participant to indicate which section they were tracking throughout the trial. Participants perform the tracking task for 12 min each run, and four runs in total. Each run followed directly after the other, except one the final one which was performed after a 12-min break. **(B)** Battelli et al. (2017) functional connectivity changes during 1 h post-stimulation. Functional connectivity scores reflect the difference between rTMS and sham sessions in the stimulated and unstimulated hemispheres. Scores outside the gray box indicate significant change in functional connectivity between rTMS and sham sessions. Data in panel **(B)** adapted from Battelli et al. (2017), copyright obtained from Elsevier and Copyright Clearance Center, licensee: Beth Israel Hospital.

Regional changes in cortical excitability have downstream impact on functionally connected circuits. Physiological measures demonstrate single pulses of TMS travel quickly to distal cortical circuits (Hallett, 2007), including to the opposite hemisphere within 30 ms of stimulation (Ilmoniemi et al., 1997; Garcia et al., 2011). Repetitive trains of rTMS propagate through functionally connected neural systems via callosal and cortico-cortical pathways (Paus et al., 1997; Walsh and Cowey, 2000; Hallett, 2007; Romei et al., 2008; Bestmann and Feredoes, 2013; Dayan et al., 2013). Examining the functional connectivity changes after LF-rTMS to the parietal cortex, Battelli et al. (2017) discovered three stages of critical changes in the dorsal attention network (Figure 1). First, an acute decrease in connectivity between homotopic regions and inter-regional activity correlation within the dorsal attention network. Then, at 36 min post-stimulation, a normalization of the activity, returning to baseline. Finally, a late 50-min increase in connectivity between the unstimulated parietal cortex, frontal eye fields (FEF) and human MT+ was observed (Figure 1B). These dynamic changes across time demonstrate not only the durable effects of LF-rTMS, but also the need to extend data sampling beyond the time-point when behavior seemingly returns to baseline. This might correspond to a crucial timepoint where compensatory effects help recovery, potentially mimicking a post-stroke response in the brain (Silasi and Murphy, 2014).

In a study comparing LF-rTMS and theta burst stimulation over the FEF on saccade latency, an increase in latency lasted for 30 min after theta burst stimulation, whereas latency returned to baseline within 12 min after LF-rTMS (Nyffeler et al., 2006). Once at baseline, saccade latency after LF-rTMS was no longer recorded. A later change in latency may have been demonstrated with longer recording sessions post-stimulation, like the later changes in functional connectivity found by Battelli et al. (2017).

## Prolonged Effects of LF-rTMS With Multiple Sessions

**FIGURE 2:**
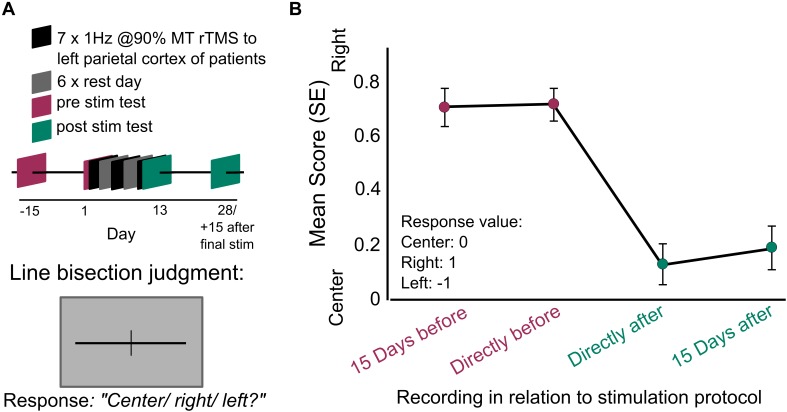
Prolonged behavioral benefit following multiple session of LF-rTMS in the parietal cortex. **(A)** Brighina et al. (2003) methods: patients with ischemic stroke to right hemisphere received seven session of 1 Hz rTMS to the healthy left hemisphere for 10 min at 90% individual motor threshold. The sessions were delivered every other day for 2 weeks. At four different recording sessions spanning before and after the seven stimulation sessions, the patients performed a line-bisection judgment. Participants were presented with lines which had been previously bisected and asked to determine if the bisection was at center, rightward of center, or leftward of center. **(B)** Brighina et al. (2003), line bisection judgment recorded: (1) 15 days before the first stimulation day, (2) directly before the first stimulation, (3) directly after the last stimulation, (4) 15 days after last stimulation day. Judgments are scored at zero for correct responses, positive values for rightward errors and negative values for leftward values. Rightward errors are highly indicative of left visual field neglect. Error bars indicate SEM. Data from **(B)** adapted from Brighina et al. (2003), copyright obtained from Rightslink^®^ and Copyright Clearance Center, licensee: Beth Israel Hospital.

Given the prolonged stimulation effects following one session, one might ask whether the beneficial effect of LF-rTMS can be extended further to become sustained across months, and thus indicating LTP and/or LTD like features. We therefore look toward the long-lasting effect of LF-rTMS after multiple sessions. Following the finding that rTMS to the healthy hemisphere of a stroke patient results in behavioral improvement contralateral to the stroke hemisphere, multi-session studies have been performed to extend these effects (Brighina et al., 2003; Khedr et al., 2005; Fregni et al., 2006; Demirtas-Tatlidede et al., 2015). Patients with chronic stroke who received 5 days of LF-rTMS to the unaffected motor cortex improved motor abilities in their stroke-affected hand lasting up to 2 weeks post-stimulation (Fregni et al., 2006). A following study, which increased the stimulation protocol to 10 days, found 4 weeks of post-stimulation improvement in the stroke-affected hand, accompanied by an increase in transcallosal fractional anisotropy values (Demirtas-Tatlidede et al., 2015). In another study patients after acute ischemic stroke received 10 days of stimulation, and motor effects of LF-rTMS lasted 10 days post-stimulation (Khedr et al., 2005). A study which focused on rehabilitation with aphasia patients found positive stimulation effects lasting 8 months following 10 days of LF-rTMS (Naeser et al., 2005). Furthermore, patients with left hemispatial neglect experienced amelioration of their visuospatial deficits which lasted 15 days following seven session of LF-rTMS over 2 weeks (Brighina et al., 2003; Figure 2). Likewise, the antidepressant effects of LF-rTMS have also been tested using multi-session protocols. LF-rTMS to right DLPFC over ten sessions resulted in at least 2 weeks of antidepressant effects post-stimulation (Stern et al., 2007). Although multi-session studies regularly examine HF- and LF-rTMS protocols on antidepressant outcomes (e.g., Fitzgerald et al., 2003), very few follow-up with patients weeks after stimulation (Cao et al., 2018).

These studies demonstrate the potential for multi-session stimulation protocols as an aid to therapeutic intervention with patients. However, like single session protocols, more systematic evaluation of protocol design is necessary (e.g., Robertson et al., 2003). For example, in some protocols, stimulation is performed alongside behavioral therapy (Khedr et al., 2005), whereas other protocols stopped therapy during the experiment (Brighina et al., 2003). Behavioral training has been demonstrated as an effective tool in improving motor function in stroke patients (Liepert et al., 2000). The mix of training and stimulation is more effective than training or stimulation alone (Krause and Kadosh, 2013; Looi et al., 2017; Brem et al., 2018), suggesting the combination could be a very powerful therapeutic tool. Furthermore, the incorporation of the methods employed to elongate the efficacy of rTMS in one session could positively impact the current designs in multi-session stimulation (see section “State Dependency” below).

Physiology studies on multi-day protocols are yet to study a similar timeline to that described in humans. Markers associated with neuroplasticity were examined in rats 3 days after a 10-day protocol of either HF- or LF-rTMS (Gersner et al., 2011). It was found that HF-rTMS significantly increased neuroplasticity markers in awake rats, but there was no such impact following LF-rTMS. However, a recording at 3 days may not have been sufficient to capture the longer-lasting effects of LF-rTMS suggested by long-lasting effects found in patients (Brighina et al., 2003; Fregni et al., 2006; Stern et al., 2007; Demirtas-Tatlidede et al., 2015). To conclude, while there is a clear evidence of the benefit of multiple sessions of LF-rTMS upon behavior in pathological conditions, there are much fewer studies on benefits in the healthy population, despite empirical data show the potential long-term potentiation LF-rTMS might have on cognitive performance in the normal population (Luber and Lisanby, 2014).

## Prolonging LF-rTMS: State Dependency

Having demonstrated prolonged LF-rTMS effects from single- and multiple-session protocols, we now examine the variables which prescribe effective stimulation. By definition, brain stimulation is expected to have an effect on brain state, resulting in neurological impact, and potential behavioral alterations. Therefore, the state of the brain at the time of intervention may also influence the impact of stimulation. For example, delayed effects of rTMS have been shown to depend on muscular-exertion during stimulation in the motor cortex (Ziemann et al., 2008; Todd et al., 2009). The concept of stimulation and state dependency has been well discussed in previous reviews (see Silvanto and Pascual-Leone, 2008; Romei et al., 2016b). Here, we specifically highlight prolonged stimulation effects following the control of brain state.

The common methods to control brain state prior to brain stimulation include: (1) priming the brain with cortical stimulation, (2) pharmacological intervention, (3) behavioral task. The method which has been employed to successfully prolong brain stimulation effects is priming. Simply, priming involves applying brain stimulation to control neural activity prior to another brain stimulation to affect the primed region. Priming leverages off of meta-plasticity which is a persistent form of plasticity where the history of synaptic activity predicts lasting synaptic change (Abraham and Bear, 1996; Ridding and Ziemann, 2010). One prominent study in the motor domain demonstrated HF-rTMS followed by LF-rTMS to M1 resulted in lasting depression of motor evoked potential for up to 60 min post-stimulation, whereas LF-rTMS alone returned to baseline within 10 min (Iyer et al., 2003). Pre-conditioning the motor cortex with transcranial direct current stimulation (tDCS) followed by LF-rTMS can also elongate the effect of LF-rTMS on motor cortex excitability (Siebner et al., 2004).

Pharmacological intervention and behavioral tasks have also been demonstrated to influence the impact of stimulation (Silvanto et al., 2007; Ziemann et al., 2015). Using pharmacological intervention, stimulation induced LTP-like plasticity can be shifted to LTD-like plasticity by administering a partial NMDAR antagonist D-cycloserine prior to stimulation (Nitsche et al., 2004). Comparably, behavioral color adaptation can change the effect of TMS on the visual cortex causing usually colorless visual phosphenes (little blips of light; Marg and Rudiak, 1994) to appear in the adaptation color (Silvanto et al., 2007). Although priming has demonstrated the dependency of prolonged brain stimulation effects on brain state, to our knowledge, pharmacological intervention and behavioral tasks have not been used to prolong brain stimulation. Combined CNS-active drugs and brain stimulation interventions may have been overlooked as the two are usually paired to understanding the separate roles of drug and stimulation protocols on cortical plasticity (e.g., Liebetanz et al., 2002; Nitsche et al., 2004). Likewise, in the study of brain-behavior relationships using TMS, chronometric studies (single TMS pulses delivered at the onset of the stimuli) have always been considered more appropriate to study *acute* causal relationships (e.g., Silvanto et al., 2007). However, with the evidence of priming prolonging LF-rTMS effects, stimulation coupled with pharmacological interventions or behavioral tasks could help exert sustained beneficial effects, a desirable outcome when working with clinical population.

## The Effect of Individual Differences

Thus-far our review has outlined the potential of LF-rTMS in producing long-lasting behavioral change, however, there is also a high variability in study outcome, which can be explained through individual differences (Ridding and Ziemann, 2010; Krause and Cohen Kadosh, 2014). A basic method to control for individual subjects’ variability is to measure either individual phosphenes or motor threshold to set a stimulation intensity for a subsequent experiment (Pascual-Leone et al., 1992; Marg and Rudiak, 1994). Although this method is useful when stimulating at an individualized level within their own target region (e.g., using phosphenes threshold for a subsequent visual task), there is mixed evidence as to whether they are informative of one another (Deblieck et al., 2008) as some studies have found no correlation between motor and phosphene thresholds (Stewart et al., 2001; Boroojerdi et al., 2002; Antal et al., 2003). There is also high variability among individual anatomical brain structures, and fMRI-guided neuro-navigated TMS can help determine brain targets with good precision (Sack et al., 2009).

Other individual differences are less easy to circumvent. For example, a review by Ridding and Ziemann (2010) highlights an increase in plasticity in females following brain stimulation, but a decrease in plasticity following stimulation with age across the whole population. Furthermore, some studies find highly varied response to stimulation and have discovered the variance is due to individual baseline task performance (Santarnecchi et al., 2016). Santarnecchi et al. (2016) divided participants into fast and slow performers following a complex logic task. They found only slow performers became significantly faster after stimulation relative to baseline (Hedges’ *g* effect size = 0.80), while fast performers did not show any change in performance speed (Hedges’ *g* effect size = 0.21). One way to standardize pre-stimulation baseline is to threshold performance capability well below ceiling (for example, 75% task accuracy). Thresholding performance can reduce intra-subject variability and increase potential for stimulation effects (illustrated by Santarnecchi et al., 2016). To illustrate, we calculated the effect sizes of two studies with comparable stimulation protocols and measures, and found the effect size was larger and more robust when all participants were tested at threshold (Battelli et al., 2009; Hedges’ *G* = 0.86) than in the study where a thresholding procedure was not employed (Edwards et al., 2017; Hedges’ *G* = 0.63). Thus, testing subjects at their performance threshold might reduce variability and boost stimulation effects.

## Future Directions

In light of the potential clinical application of LF-rTMS, the durability of the positive outcomes should be well understood, indicating a need for more physiological studies. Some ground-breaking work has already been performed on the effects of stimulation in non-human primates (NHPs) and cats (Valero-Cabré et al., 2007; Bolzoni et al., 2013; Krause et al., 2017). Valero-Cabré et al. (2007) studied the brain metabolism in anesthetized cats post-stimulation and found a decrease in ^14^C-2DG uptake for 30–60 min following LF-rTMS stimulation. More recently, using anodal transcranial direct current stimulation (a-tDCS) in anesthetized cats, a network-wide boost in neuronal activation was found hours after stimulation (Bolzoni et al., 2013). In contrast, a-tDCS in awake behaving NHPs was found to effect low-frequency brain oscillations, not firing rate, and accelerates association learning (Krause et al., 2017). These findings create a foundation for future physiology studies, which in turn can inform human stimulation protocols. For example, confirming the hypothesized mechanisms behind state-dependent stimulation protocols would be incredibly valuable, and aid individualization of protocols.

The purpose of this review was restrained to the long-lasting effects of LF-rTMS, however, the inclusion of other brain stimulation techniques has been necessary to better illustrate meta-plasticity and priming. HF-rTMS and transcranial electric stimulation (tES) also show huge promise in prompting lasting plasticity in the brain, and our future directions would not be complete without the suggestion of probing other stimulation methods. With that, we would like to highlight a few examples below.

## Prolonged Behavioral and Brain Effects After tES and HF-rTMS

Transcranial electric stimulation techniques include transcranial direct current stimulation (tDCS), transcranial alternating current stimulation (tACS), and transcranial random noise stimulation (tRNS), all of which have demonstrated lasting behavioral and/or physiological effects in the visual (Herpich et al., 2018), parietal (Cappelletti et al., 2013), and motor cortex (Kuo et al., 2013). Kasten et al. (2016) found a sustained enhancement of alpha power 70-min after individual alpha frequency was presented to the occipital cortex using tACS, indicating the selectivity of the effect of tACS. Moreover, tRNS applied to the motor cortex and visual cortex has induced consistent excitability recorded through increase motor evoked potentials and decrease phosphene threshold, respectively, which lasted 60 min post-stimulation (Terney et al., 2008; Herpich et al., 2018). Furthermore, multi-session tRNS to bilateral dorsolateral prefrontal cortex resulted in a boost in mental arithmetic 6 months post-stimulation, also correlated with an increase in activity over left DLPFC (Snowball et al., 2013). Altogether these studies indicate that tRNS might increase excitability and open up a *critical* window during which the cortex might be more plastic and responsive to treatment.

Transcranial magnetic stimulation protocols (other than LF-rTMS) have also shown potential for prolonging post-stimulation effects. For example, HF-rTMS over the FEF has resulted in a 60-min increase in saccade latency (Nyffeler et al., 2006), and effects on functional connectivity lasting 30–40 min (Rizk et al., 2013). HF-rTMS over the rat cortex has demonstrated local neural activity impact lasting 160 min post-stimulation (Hoppenrath and Funke, 2013). Finally, an interesting recent stimulation method, namely cortico-cortical paired association (ccPAS), has been found to strengthen reentrant connectivity 30 and 60 min after stimulation, specifically from V5 to V1 (Romei et al., 2016a). ccPAS has the potential of inducing selective pathway-specific changes with a multi-coil approach, and enhance the precision and selectivity of the effect (Chiappini et al., 2018). This has clear potential for individualized clinical interventions. Thus, accumulating evidence for positive lasting behavioral and brain activity changes following stimulation indicates a real possibility for these stimulation protocols in rehabilitation therapies.

## Concluding Remarks

This review highlights prolonged neuromodulatory effects on brain dynamics and behavior of humans and animals following non-invasive cortical stimulation. Harnessing these long-term effects should be a high-priority if brain stimulation is to be a powerful aid in rehabilitation. We expect physiology experiments will be a driving force in honing stimulation protocols to better exploit long-term neurological and behavioral benefits.

## Author Contributions

GE, EG, and LB wrote the manuscript. SA, FH, FC, DP, and ST edited the manuscript.

## Conflict of Interest Statement

The authors declare that the research was conducted in the absence of any commercial or financial relationships that could be construed as a potential conflict of interest. The reviewer LR declared a shared affiliation, though no other collaboration, with several of the authors, FC and DP, to the handling Editor.
